# The use of virtual reality technology among women undergoing intrauterine insemination: a randomized controlled study

**DOI:** 10.1038/s41598-025-32620-8

**Published:** 2026-02-05

**Authors:** Ghazaleh Gholami, Tahereh Behroozi lak, Roghieh Bayrami, Maryam Mesgarzadeh

**Affiliations:** 1 Kosar Women’s Comprehensive Hospital, Urmia University of Medical Sciences, Urmia, Iran; 2 Reproductive Health Research Center, Clinical Research Institute, Urmia University of Medical Sciences, Urmia, Iran; 3 Department of Midwifery, Faculty of Nursing and Midwifery, Urmia University of Medical Sciences, Urmia, Iran

**Keywords:** Infertility, Virtual reality, Intrauterine insemination, Anxiety, Depression, Worry, Psychology, Health care, Medical research

## Abstract

Women diagnosed with infertility often experience elevated psychological distress both before and during treatment, which can adversely affect clinical pregnancy rates. The aim of the present study was to investigate the role of virtual reality technology in decreasing psychological distress and increasing pregnancy rates in women diagnosed with infertility undergoing intrauterine insemination (IUI). This randomised controlled trial study was conducted at Kosar University Hospital in Urmia, Iran, in 2024, involving 114 women diagnosed with infertility. Participants were randomly allocated to either the intervention group (n = 57), which viewed a selected video with 360-degree virtual reality glasses for 20 min before and 10 min during the IUI procedure, or the control group (n = 57), which received standard care. Psychological outcomes were assessed using the Hospital Anxiety and Depression Scale (HADS) and the Pennsylvania State Worry Questionnaire (PSWQ). The clinical pregnancy rates, a secondary outcome, was evaluated 14 days post-IUI using a Beta hCG test. Results indicated that the intervention group exhibited significantly lower median scores (interquartile range) for worry, anxiety, and depression compared to the control group (*p* < 0.001). Furthermore, the odds of a positive pregnancy test were 2.76 times higher in the intervention group (95% CI, *p* < 0.01). These findings suggest that virtual reality technology is an effective intervention for reducing psychological distress and improving pregnancy outcomes in women undergoing IUI.

*Trial Registration*: The study was registered in the Iranian Registry of Clinical Trials (IRCT Id: **IRCT20231013059702N2** Registration date: **2024-02-11**).

## Introduction

Infertility is not classified as a disease, but it and its treatment can cause various psychological and emotional disorders including increased stress, depression, and anxiety^1^. An overview of systematic reviews and meta-analyses showed that the current prevalence of infertility in Iran is 13.2%^2^. Although male and female factor infertility occur at nearly equal rates^3^, women often experience greater emotional distress associated with infertility than men. Intrauterine insemination (IUI), a common treatment option, can be an emotionally and physically demanding process for patients. It typically involves a series of steps, including the use of prescription medications, frequent vaginal ultrasounds, and semen analysis, all of which can contribute to heightened stress and emotional strain^4^. Prolonged exposure to these emotions can even lead to the development of depression over time^5–7^.

It is crucial to emphasise that moderate to high levels of anxiety and depression can significantly diminish a patient’s willingness to persist with treatment. Additionally, the prolonged duration and substantial costs associated with infertility diagnosis and treatment can impose considerable psychological strain on patients. These factors not only exacerbate psychological symptoms but may also adversely affect treatment outcomes^8–11^. Furthermore, as patients approach the end of their treatment journey, levels of anxiety, stress, and depression often intensify, potentially leading to a higher incidence of negative outcomes^12^. A study by Massarotti et al. highlighted that when infertility is attributed solely to female factors, women are more likely than men to experience elevated rates of anxiety and distress both before and during treatment^13^. Among the most effective non-pharmacological interventions for alleviating anxiety, depression, and pain are behavioural therapy techniques, including thought diversion strategies^14,15^. One such intervention is virtual reality (VR) therapy, which aims to reduce anxiety and depression and may subsequently decrease pain perception^16^.

However, the effectiveness of VR technology in medical settings remains a topic of debate. For example, a study by Melser et al. demonstrated that the use of VR glasses during amniocentesis significantly reduced the amount of pain perceived by women, though it did not significantly alleviate anxiety levels^17^. In contrast, a study by Jahani Shourab et al. found that VR glasses significantly reduced anxiety in women undergoing episiotomy repair^18^.

The aim of the present study was to investigate the role of VR technology in decreasing psychological stress and increasing pregnancy rate in women diagnosed with infertility undergoing IUI.

## Methods

### Study design and setting

This single-blind randomised controlled trial was conducted among women diagnosed with infertility undergoing IUI at Kosar University Hospital between September 2023 and January 2024. The reporting of the randomised trial followed the guidelines specified in the CONSORT 2025 statement^19^.

### Sample size and Participants

The sample size was calculated using the mean anxiety scores reported in a study by Murphy et al.^20^, where the intervention group had a mean score of 29.3 ± 8.4 and the control group had a mean score of 33.76 ± 10.7. With a 90% confidence interval (Z1-α/2 = 1.64) and a test power of 80% (Z1-β = 0.84), the minimum sample size was determined to be 57 women per group.

$$n = \frac{{\left( {Z_{{1 - \frac{\alpha }{2}}} + Z_{1 - \beta } } \right)^{2} \times \left( {S_{1}^{2} + S_{2}^{2} } \right)}}{{\left( {\overline{X}_{1} - \overline{X}_{2} } \right)^{2} }}$$

The inclusion criteria for the study were as follows: consent to participate in the study, first-time experience with IUI treatment, and diagnosis of female-factor infertility. The exclusion criteria included: a history of psychological disorders, addiction to cigarettes, alcohol, or opium, self-reported use of psychotropic medications, and self-reported experience of any psychological crisis within the past three months (e.g., death of a close relative), and presence of any disease or disability that could interfere with the study’s objectives.

### Recruitment of participants

**Fig. 1:**
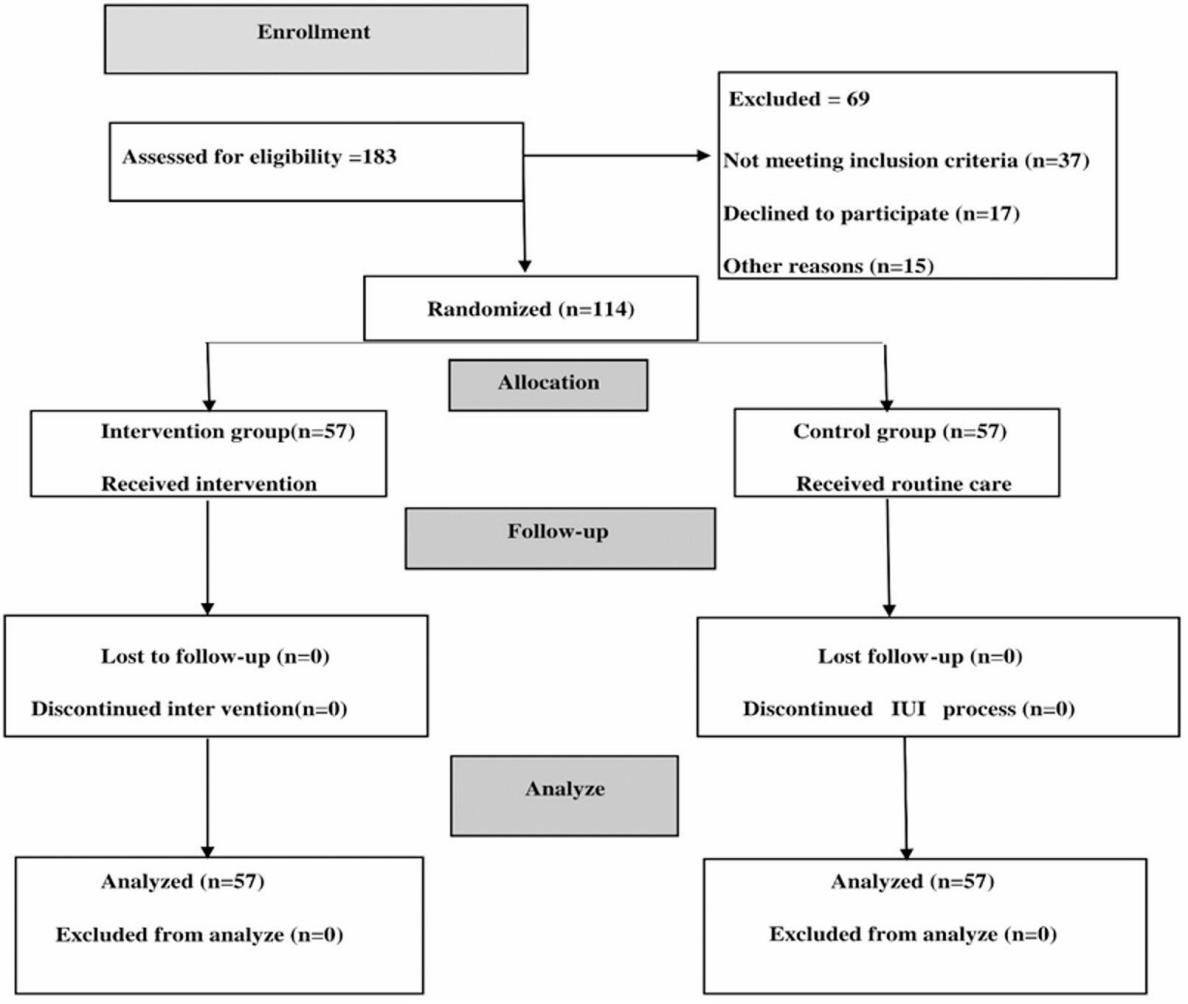
CONSORT diagram describing study enrolment and allocation.

The researcher (Gh.Gh) visited the infertility clinic every weekday to identify eligible women who were candidates for IUI using a convenience sampling method and they were then asked to participate in the present study. Those who agreed were screened for inclusion criteria. The study’s objectives and procedures were explained to eligible participants, and written informed consent was obtained from all individuals prior to enrolment. Participants were randomly allocated in a 1:1 ratio to either a VR (intervention group) or routine treatment (control) group. For randomisation, 114 cards were prepared, laballed with a number from 1 to 114 and placed in opaque envelopes. The selected card number determined the participant’s group. Odd-numbered participant records were assigned to the intervention group, and even-numbered participant records were assigned to the control group. To prevent bias, randomisation was performed independently from individuals involved in data analysis and sampling. The data analyst was blinded in this trial. Sample selection and randomisation are explained in Fig. 1.

## Measurements

### Demographic information

A sociodemographic questionnaire (11 items) was designed by the research team. The content validity of the questionnaire was assessed using feedback from 10 faculty members of Urmia University of Medical Sciences (for example, how to classify income). Necessary corrections were made based on the feedback received.

### Outcome variables

The primary outcome variables included measures of hospital anxiety and depression, as well as worry, assessed using the Hospital Anxiety and Depression Scale (HADS)^21^ and the Pennsylvania State Worry Questionnaire (PSWQ)^22^, respectively. The secondary outcome variable was the clinical pregnancy rate, confirmed via a blood tes (Beta hCG test).

The HADS is a 14-item self-report questionnaire designed to assess symptoms of anxiety and depression. It consists of two seven-item subscales: one measuring anxiety (HADS-A) and the other measuring depression (HADS-D). Scores on each subscale range from 0 to 21, with higher scores indicating more severe symptoms. The questionnaire employs a Likert scale ranging from 0 to 3 to capture participants’ perceptions. Items 1, 3, 5, 6, 8, 10, 11, and 13 are reverse-scored^21^. The Iranian version of the HADS was validated by Montazeri et al. in 2003, demonstrating its reliability and validity for use in Iranian populations. The Cronbach’s alpha coefficient for the questionnaire was reported to be greater than 0.7, indicating good internal consistency and reliability of the instrument^23^.

The PSWQ was developed to assess the trait of worry^22^. It comprises 16 items, each rated on a 1 to 5-point scale, resulting in a total score ranging from 16 to 80. Scores above 44 indicate a clinically significant level of worry, with 44–62 reflecting a moderate level and 63–80 representing a high level of worry. The Iranian version of the PSWQ was validated by Salarifar et al. in 2012, demonstrating its reliability and validity for use in Iranian populations^24^.

### Intervention

Initially, the researcher downloaded three videos depicting natural landscapes onto a mobile device. The selection criteria prioritised video clarity and attractiveness, a duration of 10–15 min, and the presence of only natural sounds within the depicted landscapes. The first video showcased a forest scene with lush vegetation and a narrow river, accompanied by the sounds of birdsong and the gentle flow of the stream. The second video featured a seashore with a clear blue sky and sea waves, capturing the sounds of the waves. The third video portrayed a green mountain covered in flowers and shrubs, highlighting a beautiful mountain stream with subtle birdsong. During the intervention, the mobile phone containing the videos was integrated with a headset incorporating 360-degree VR glasses.

Women in the intervention group wore the VR headset for 20 min prior to and during the IUI procedure, which lasted a maximum of 10 min. Participants remained in a supine position on the gynaecology bed throughout the intervention. Women viewed one of the videos, selected based on their preference. The videos were set to automatically replay upon completion. The control group received usual care before, during, and after the procedure. All participants in both groups received routine care (post-IUI recommendations included: rest, a healthy diet, stress management , avoiding strenuous activities, heavy lifting, and hot baths or saunas, and adhering to the doctor’s prescribed medications and follow-up appointments).

### Data collection

On the day of the IUI procedure, participants from both groups completed the questionnaires in a calm and quiet environment 30 min prior to the procedure. After the IUI process, participants were transferred to the recovery room, where they completed the same questionnaires again 30 min post-procedure. In the final phase, approximately 14 days after the IUI, serum Beta hCG levels were measured to assess the clinical pregnancy rate, and the occurrence of pregnancy was documented for both groups.

### Data analysis

Descriptive statistics, including mean, standard deviation, frequency, and percentage, were used for data analysis. The normal distribution of data was first evaluated using the Kolmogorov–Smirnov test. Given the normal data distribution, independent t-tests, Chi-square tests, and Fisher’s exact test were used. The Mann–Whitney U-test was used to compare non-normally distributed variables between the two groups. Statistical analysis was performed using SPSS16. A *p*-value of < 0.05 was considered statistically significant.

### Ethical considerations

This study received approval from the Ethics Committee under reference number IR.UMSU.REC.1402.321 and was registered with the IRCT. Prior to participation**, **the study’s objectives and procedures were clearly explained to all participants, and written informed consent was obtained. Participants were assured of the confidentiality of their information and were informed of their right to withdraw from the study at any time. It is important to note that both groups of women received standard medical treatment, and the intervention was implemented without interfering with their routine medical care.

## Results

### Baseline characteristics

The present study included 114 women diagnosed with infertility. The mean age (standard deviation) of participants and their spouses was 32.14 ± 4.66 and 32.49 ± 5.85 in the intervention group and 32.46 ± 4.64 and 32.14 ± 6.17 in the control group, respectively. The mean duration of infertility (standard deviation) was 4.58 ± 2.36 and 4.53 ± 2.35 in the intervention and control group, respectively. The majority of women in both groups were homemakers and reported an inadequate family income. No significant differences were observed between the groups in terms of demographic characteristics (*p* > 0.05) (Table 1).

**Table 1:** Socio-demographic characteristics of participants.

Variable		Intervention group(*N* = 57)	Control group(N = 57)	*P*-value
		Mean ± SD	Mean ± SD	
Age of women (year)		32.14 ± 4.662	32.46 ± 4.645	*P = 0.718
Age of spouse (year)		32.49 ± 5.856	32.14 ± 6.177	*P = 0.756
Duration of marriage (year)		6.04 ± 2.822	5.67 ± 2.452	*P = 0.458
Duration of infertility (year)		4.58 ± 2.360	4.53 ± 2.354	*P = 0.905
BMI		25.95 ± 2.51	26.33 ± 2.44	*P = 0.423

		n(%)	n(%)	
Education level of women	Illiterate Under the diploma Diploma Post graduate Bachelor degree Master degree and above	1 (1.8) 15(26.3) 20(35.1) 9(15.8) 11(19.3) 1(1.8)	0(0) 19(33.3) 18(31.6) 8(14) 9(15.8) 3(5.3)	**P = 0.725
Spouse’s level of education	Illiterate Under the diploma Diploma Post graduate Bachelor degree Master degree and above	1(1.8) 15(26.3) 11(19.3) 13(22.8) 13(22.8) 4(7)	1(1.8) 16(28.1) 16(28.1) 7(12.3) 11(19.3) 6(10.5)	**P = 0.650
Employment status of women	Housekeeper Employed	41(71.9) 16(28.1)	37(64.9) 20(35.1)	***P = 0.546
Spouse’s employment status	Self-employed Employed	41(71.9) 16(28.1)	41(71.9) 16(28.1)	***P = 1
Family income	Inadequate Relatively Adequate	33(57.9) 22(38.6) 2(3.5)	30(52.6) 27(47.4) 0(0)	**P = 0.265
Place of residence of the couple	City The village	36(63.2) 21(36.8)	38(66.7) 19(33.3)	***P = 0.845

*Independent t-test, **Fisher’s exact test, ***Chi-squared test.

### Primary outcomes

According to the independent t-test, a statistically significant difference was found in worry scores between the two groups following the intervention (*p* < 0.001). Furthermore, the Mann–Whitney U-test revealed significant differences between the groups in the median scores of the HADS anxiety subscale, HADS depression subscale, and total hospital anxiety and depression after the intervention (*p* < 0.001) (Table 2).

**Table2:** Comparison of mean scores of worries, and median scores of hospital anxiety and depression at the time before and after intervention in the two intervention and control groups.

Variable		Intervention group	Control group	*P*-value*
		Med( IQR)	Med (IQR)	
HADS-A	Pre-intervention Post-intervention	15(13–16) 8(7–9)	14 (13–16) 16 (14–17)	*p = 0.37 *p < 0.001
HADS-D	Pre-intervention Post-intervention	14 (13–16) 9(8–10)	15(13–16) 16(15–17)	*p = 0.48 *p < 0.001
HADS	Pre-intervention Post-intervention	29(27–31) 16(14–18)	29(32–26) 32(34–29)	*p = 0.99 *p < 0.001

		Mean ± SD	Mean ± SD	
Worry	Pre-intervention Post-intervention	61.40 ± 4.68 52.22 ± 4.44	61.24 ± 4.42 64.26 ± 3.76	**p = 0.853 *p < 0.001*

*Mann–Whitney U test.

**Independent t- test.

### Secondary outcome

The odds ratio (OR) for a positive pregnancy test 14 days post-intervention was 2.76 (95% CI; *p* < 0.01) in the intervention group, indicating that participants receiving the intervention were 2.76 times more likely to achieve pregnancy compared to those in the control group (Table 3).

**Table 3:** Comparison of Beta hCG results in two intervention and control groups.

Variable	Intervention group	Control group	*P* value	Odds ratio: 0.95% CI
	n(%)	n(%)		
Beta hCG (Positive)	14(70)	6(30)	*p = 0.049	2.76
Beta hCG (Negative)	43(45.7)	51(54.3)		

*Chi–Squared test.

## Discussion

The findings of the present study highlight the promising potential of VR technology in reducing symptoms of hospital anxiety and depression, alleviating worry, and improving pregnancy rates among women undergoing IUI treatment. The median scores for hospital anxiety and depression, as well as the mean score for worry, were significantly lower in the VR group compared to the control group. Furthermore, participants in the intervention group were 2.76 times more likely to achieve a pregnancy compared to those in the control group.

In this study, both groups initially exhibited high median scores for anxiety and depression. Previous research^25,26^ has demonstrated that infertility and the use of assisted reproductive technologies can contribute to increased levels of anxiety and depression in women and their spouses. Notably, the risk of experiencing these psychological challenges appears to be higher among women^27^. Virtual reality is an innovative strategy that involves the use of computer-generated, three-dimensional environments to create immersive experiences. As a cognitive- behavioural intervention tool, VR has been increasingly utilised to address a variety of medical conditions. It has shown particular promise in managing common symptoms such as anxiety, depression, and pain, which are prevalent across numerous health challenges^28,29^. The study by Garcia Pombo et al. (2023), the use of VR glasses during the embryo transfer process was found to reduce anxiety, aligns with the findings of the present study. Although a higher pregnancy rate was observed in their intervention group, the difference was not statistically significant^30^. Similarly, Almedhesh et al. (2022) concluded that VR glasses can reduce stress and anxiety in women undergoing caesarean section, a result consistent with the present study^31^. Further supporting our results**,** Chan et al. (2020) demonstrated that the use of VR technology can alleviate anxiety and depression in women undergoing gynaecological procedures^32^. Zhang et al. (2019) also found VR to be effective in reducing anxiety levels among women undergoing dilation and curettage. Their findings align with the results of the present study, despite differences in the statistical populations of the two studies^33^. A separate investigation by Jahani Shorab et al. (2016) revealed that VR technology can reduce anxiety and distress in women undergoing episiotomy repair, further corroborating our findings^18^.

Virtual reality possesses the unique ability to simulate immersive environments and scenarios that can evoke targeted emotional and behavioural responses, positioning it as a promising tool for addressing depression and various anxiety disorders. Research indicates that VR not only creates therapeutic settings but also serves as a powerful distraction, mentally engaging users and temporarily redirecting their focus away from distressing stimuli. This cognitive shift allows individuals to disengage from negative patterns during the immersive experience. By combining these dual mechanisms providing controlled therapeutic environments and a mental respite VR demonstrates significant potential in alleviating symptoms of anxiety and depression^34,35^.

## Conclusion

According to the results of this study, the application of VR technology was effective in reducing worry, anxiety, and depression associated with the IUI procedure in women. Furthermore, the use of VR technology was associated with an increased clinical pregnancy rate in women undergoing IUI. It is recommended that VR interventions be implemented before and during the IUI process.

### Limitations and strengths

The present study is the first to implement a VR-based intervention in the field of infertility treatment in Iran. However, several limitations should be considered. First, the study was conducted at a single centre, which may limit the generalisability of the findings to other populations. Second, the video content used in the intervention was preselected by the researchers. Third, the trial was limited by its single-blind design.

### Future research directions

Future research should evaluate a broader range of outcomes associated with VR interventions, such as fear and stress. It is also recommended that further studies incorporate a variety of video content, potentially selected by participants themselves.

## Data Availability

The data used and/or analyzed during the current study are available from the corresponding author on reasonable request.
